# Retraction: A carboxymethyl cellulose modified magnetic bentonite composite for efficient enrichment of radionuclides

**DOI:** 10.1039/d2ra90116a

**Published:** 2022-11-16

**Authors:** Rui Hu, Xuemei Ren, Guangshun Hou, Dadong Shao, Yu Gong, Xiaojun Chen, Xiaoli Tan, Xiangke Wang, Masaaki Nagatsu

**Affiliations:** Graduate School of Science and Technology, Shizuoka University Hamamatsu 432-8561 Japan; Institute of Plasma Physics, Chinese Academy of Sciences PO Box 1126 Hefei 230031 P. R. China xmren@ipp.ac.cn shaodadong@126.com +86-551-65591310 +86-551-65593308; Institute of Resources & Environment, Henan Polytechnic University Jiaozuo 454003 P. R. China; Institute of Nuclear Physics and Chemistry, China Academy of Engineering Physics Mianyang 621900 P. R. China; School of Environment and Chemical Engineering, North China Electric Power University Beijing 102206 P. R. China

## Abstract

Retraction of ‘A carboxymethyl cellulose modified magnetic bentonite composite for efficient enrichment of radionuclides’ by Rui Hu *et al.*, *RSC Adv.*, 2016, **6**, 65136–65145, https://doi.org/10.1039/C6RA10990J

The Royal Society of Chemistry, with the agreement of the named authors, hereby wholly retracts this *RSC Advances* article due to concerns with the reliability of the data in the published article.

In Fig. 1F, there are unexpected similarities within the baseline of the Fe_3_O_4_ XRD pattern in the region ∼7–12 theta, and also within the CMC-g-MB XRD pattern in the region ∼7–10 theta. An independent expert was consulted who was not satisfied with the explanation provided by the authors. The authors provided replacement XRD data, however upon review, the expert found that the new data for both Fe_3_O_4_ and CMC-g-MB appeared very different from the original manuscript.

The XRD patterns presented in Fig. 8A for CMC-g-MB-Co(ii) and CMC-g-MB-HNO_3_ appear to be identical in the region of ∼12–60 theta, but differ in the region below 12 theta. The expert was not satisfied with the authors’ explanation and concluded that there was some degree of manipulation to try and make the two XRD patterns appear to be different. The authors provided replacement XRD patterns, but the expert concluded that they were very different from those presented in the original manuscript and would affect the original discussion in the manuscript.

The 0 min and 5 min XPS spectra in Fig. S3B are identical in the regions above 102 eV, and below ∼97 eV, but differ in the region of 97–102 eV. Upon a vertical compression, the region of 97–102 eV of the 5 min spectrum is also identical to the same region of the 15 min spectrum. The authors admitted that the 5 min spectrum was computer-generated to show the expected/desired result that they would then confirm experimentally and was not intended to be used in the article. The authors carried out the XPS measurements for CMC-g-MB treated with 0, 5 and 15 min of RF plasma again, and requested to publish the replacement data. However, the expert was not satisfied with the response and raised additional concerns regarding the reliability of the raw data provided by the authors.

Furthermore, after analysing the raw data txt files and Origin files provided by the authors for Fig. 1F, 8A and S3B, the expert found that the numbers in the raw data txt files did not match the numbers given in the Origin files. The expert therefore was not satisfied that the raw data provided by the authors was reliable.

Given the significance of the concerns about the validity of the data in the published paper and the raw data, the findings presented in this paper are no longer reliable.

The other authors were contacted but did not respond.

Signed: Rui Hu, Masaaki Nagatsu

Date: 3rd November 2022

Retraction endorsed by Laura Fisher, Executive Editor, *RSC Advances*

## Supplementary Material

